# Myopic macular neovascularization treatment: A 2-year follow-up of a real-life cohort

**DOI:** 10.1007/s00417-025-06973-9

**Published:** 2025-09-26

**Authors:** Assaf Hilely, Roee Arnon, Reut Shor, Ori Segal, Justin Lerann Shad, Eran Greenbaum, Omer Trivizki, Anat Loewenstein, Gilad Rabina

**Affiliations:** 1 Department of Ophthalmology, Sackler Faculty of Medicine, Tel Aviv Sourasky Medical Center, Tel Aviv University, Tel aviv, Israel; 2 Department of Ophthalmology, Faculty of Medicine, Rabin Medical Center - Beilinson Hospital, Tel Aviv University, Petach Tiqwa, Tel Aviv, Israel; 3 Department of Ophthalmology, Sackler Faculty of Medicine, Meir Medical Center, Tel Aviv University, Kfar Saba, Israel; 4 University of California Los Angeles, Los Angeles, CA USA

**Keywords:** Myopia, Myopic choroidal neovascularization, Macular neovascularization, Anti-vascular endothelial growth factor, Pro-re-nata

## Abstract

**Purpose:**

Evaluating the impact for different number of Anti-vascular endothelial growth Factor (VEGF) injections on macular neovascularization (MNV) secondary to pathological myopia, during a 2-year follow-up period, in relation to visual outcome.

**Methods:**

This was a multicenter, retrospective study of patients with myopic MNV (mMNV) for 24 months.

**Results:**

A total of 55 patients with mMNV, with a mean age of 65.7 ± 14.5 years, met the inclusion criteria. The mean number of injections was significantly higher during the first year of follow-up with 8.78 ± 2.90 within the 1st year versus 3.45 ± 4.40 during the 2nd year (*p* = 0.037). Visual acuity (VA) remained relatively stable throughout the follow-up (*p* = 0.902), with most patients maintaining their vision or experiencing a loss of up to 5 Snellen letters. (42 patients, 76.3%, *p* < 0.001).

**Conclusion:**

During a 24-month period, there was a lack of BCVA improvement, which may be attributed to the PRN injection regimen and the relatively low number of injections administered for mMNV. These findings suggest that mMNV may be undertreated in routine practice, with suboptimal intravitreal injection frequency.

**Key messages:**

***What is known***

- The PRN injection regimen is widely used for treating myopic macular neovascularization (mMNV) due to its less aggressive nature and the potential risks of retinal break, retinal detachment, and other complications in highly myopic eyes.

***What is new***

- At the 24-month follow-up, patients with mMNV treated with anti-VEGF agents in a PRN regimen showed no significant improvement in BCVA.
- Myopic MNV may be fallacy thought of as a less aggressive pathology and therefore is undertreated and perhaps may require a much more intensive injection schedule.

## Introduction

Macular neovascularization (MNV) is a complication of pathological myopia as the 2nd most common etiology after age-related macular degeneration (AMD). Anti-vascular endothelial growth Factor (VEGF) has been widely used in treating neovascular myopic MNV (mNMV) and effectively improving and maintaining vision [1, 2]. 

Pro re nata (PRN), or “as needed,” involves administering injections only when there is evidence of active disease, such as new or worsening symptoms or signs of disease activity on imaging. It is considered common practice to treat mMNV with PRN to achieve good anatomical and functional results [3]. While PRN showed higher recurrence rates compared to the treat-and-extend (T&E) regimen, clinicians sometimes prefer it for its lower number of injections during the treatment period and for its comparable visual outcome between the two regimens [4]. 

This study aims to explore the long-term functional clinical outcomes of mMNV in response to the frequency of anti-VEGF injections.

## Methods

A retrospective, multi-center, observational study of consecutive patients with subfoveal MNV secondary to myopia, treated with anti-VEGF injections and followed by retina specialists between 01.01.2017–31.12.2021 at the ophthalmology departments of Meir Medical Center, Kfar Saba and Tel Aviv Sourasky Medical Center, Tel Aviv, Israel. The study adhered to the tenets of the Declaration of Helsinki and was approved by the Institutional Review Board (IRB) of the above-mentioned medical centers. Cases were identified by electronic medical records (EMR (of each department.

### Data collection

Patient charts were reviewed for demographics, date of diagnosis, best-corrected visual acuity (BCVA), slit-lamp examination, number and dates of anti-VEGF injections, and clinic visits. BCVA was documented and reported in Snellen fraction, which was converted into logarithm of the minimal angle of resolution (logMAR) values for statistical analysis. In cases of bilateral MNV and both eyes eligible for the study, the right eye was selected for the analysis.

### Study participants

Patients who were diagnosed with naive mMNV treated with intravitreal anti-VEGF injections, with a follow-up over a minimum period of 24 months were included.

### Treatment protocol

Patients were collectively treated with three bevacizumab loading dose, followed by a PRN protocol among the mMNV. In our country, Bevacizumab is mandated by the public healthcare system as the first-line treatment for myopic MNV; accordingly, the present retrospective study focused on this therapy.

### Inclusion criteria

mMNV was defined hyperreflective lesion with fuzzy borders at the RPE, central ellipsoid, and external limiting membrane. The overlying retina maybe only minimally elevated with or thickened with possible PED, IRF, SRF, or subretinal hemorrhage as seen on OCT or leakage on FA, with the presence of retinal changes compatible with pathologic myopia seen by fundus ophthalmology [2]. 

### Exclusion criteria

Fulfilling one of the following criteria in the study eye: (1) previous pars plana vitrectomy or any treatment for retinal detachment, (2) other concomitant macular disease (i.e. diabetic macular edema, retinal vein occlusion, macular hole, macular dystrophies, MNV due to causes other than myopia, (3) any other ocular condition compromising BCVA except the presence of cataract (i.e. advanced glaucoma, amblyopia, trauma, optic neuropathy), (4) previous treatment with intravitreal anti-VEGF injections, (5) previous macular treatment (laser photocoagulation, photodynamic therapy). All of the patients received initially 3 monthly mandatory bevacizumab injections, according to the national health guidelines. In case of inadequate response, which is defined as less than 20% decrease in baseline central macular thickness (CMT) or deteriorating BCVA after at least 3 monthly bevacizumab injections, patients are eligible to switch to alternative anti-VEGF treatment such as aflibercept or ranibizumab, at the discretion of the treating ophthalmologist. Exudative activity was identified as the occurrence of retinal hemorrhage on clinical exam, and the presence of intra- or subretinal fluid or SHRM on OCT. BCVA was assessed at baseline and at least every 6 months till the end of follow-up.

#### Statistics

All data gathered in the study was obtained from electronical medical records using the MD-Clone software (MDClone cooperation). The statistical analysis was carried out with Microsoft Excel 2017 (Microsoft Corporation, Redmond, WA) and Minitab Software, version 17 (Minitab Inc, State College, PA). Quantitative variables were described as mean ± standard deviation (SD). Categorical variables were described as absolute and relative frequencies. The Paired T test, the Student’s T-test and Kruskal-Wallis tests were used for the comparison of continuous outcomes for paired and non-paired groups. For the comparison of categorial outcomes of paired and un-paired groups the McNemar’s test and Chi-Square test were applied. A P-value of less than 0.05 was considered statistically significant.

## Results

Fifty-five eyes from 55 patients were identified with mMNV met the inclusion criteria and included in this study. The mean age was 65.7 ± 14.5 years old Females comprised the majority of the group with 58.2% of the patients. (Table 1)

**Table 1:** Baseline characteristic of the myopic patients, number of intravitreal injections per year, and BCVA at baseline and final visits

*N*	55
Female Gender	32 (58.2%)
Age (years)	65.7 ± 14.5
Mean number of anti VEGF Injections during 1 st year of follow-up	8.78 ± 2.90
Mean number of anti VEGF Injections during 2nd year of follow-up	3.45 ± 4.40
Baseline BCVA (LogMar)	0.44 ± 0.37
Final BCVA (LogMar)	0.43 ± 0.41

*MNV*- Macular neovascularization

*VEGF*- Vascular endothelial growth factor

*BCVA*- Best corrected visual acuity

**Fig. 1:**
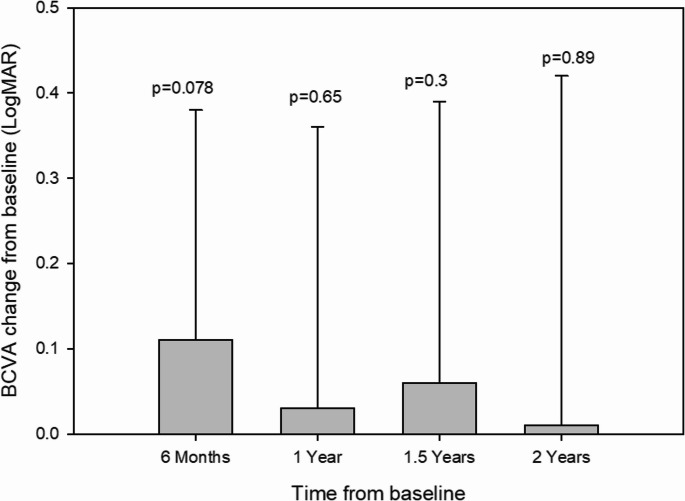
The mean best-corrected visual acuity (BCVA), expressedin logarithm of the minimum angle of resolution (logMAR) units, was recorded at baseline and at six-month intervals up to 24 months of follow-up

Following six months of therapy, the mean best-corrected visual acuity (BCVA) exhibited a trend toward improvement;however, fluctuations in BCVA, characterized by intermittent gains and losses, were observed throughout the follow-up period. At the conclusion of thetwo-year follow-up, mean BCVA had returned to a level comparable to baseline. (Fig. 1).

Most of the patients (76.3%) remain stable or lost up to 5 Snellen letters (*p* < 0.001, Table 2).

**Table 2:** Visual acuity loss during the first and second years of follow-up

Snellen Letters Lost	0–5	6–10	> 10	*p*-Value
Number of patients - N, (%)	42 (76.3%)	3 (5.5%)	10 (18.2%)	*P* < 0.001

The mean number of injections was significantly higher during the first year of follow-up with 8.78 ± 2.90 within the 1 st year versus 3.45 ± 4.40 during the 2nd year (*p* = 0.037, Table 1).

**Fig. 2:**
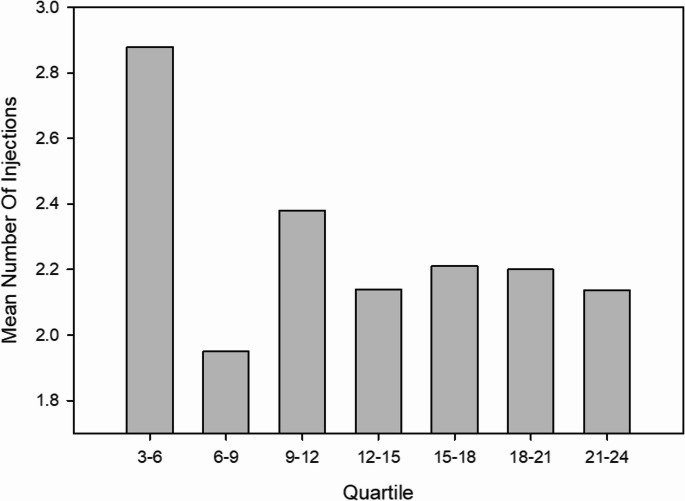
Mean number of intravitreal injections administered per patient during each quarterly period over the study duration

Patients showed a decrease in the number of injections after the initial 3 months of monthly treatment, with a more variable trajectory during the follow-up period. (Fig. 2)

**Fig. 3:**
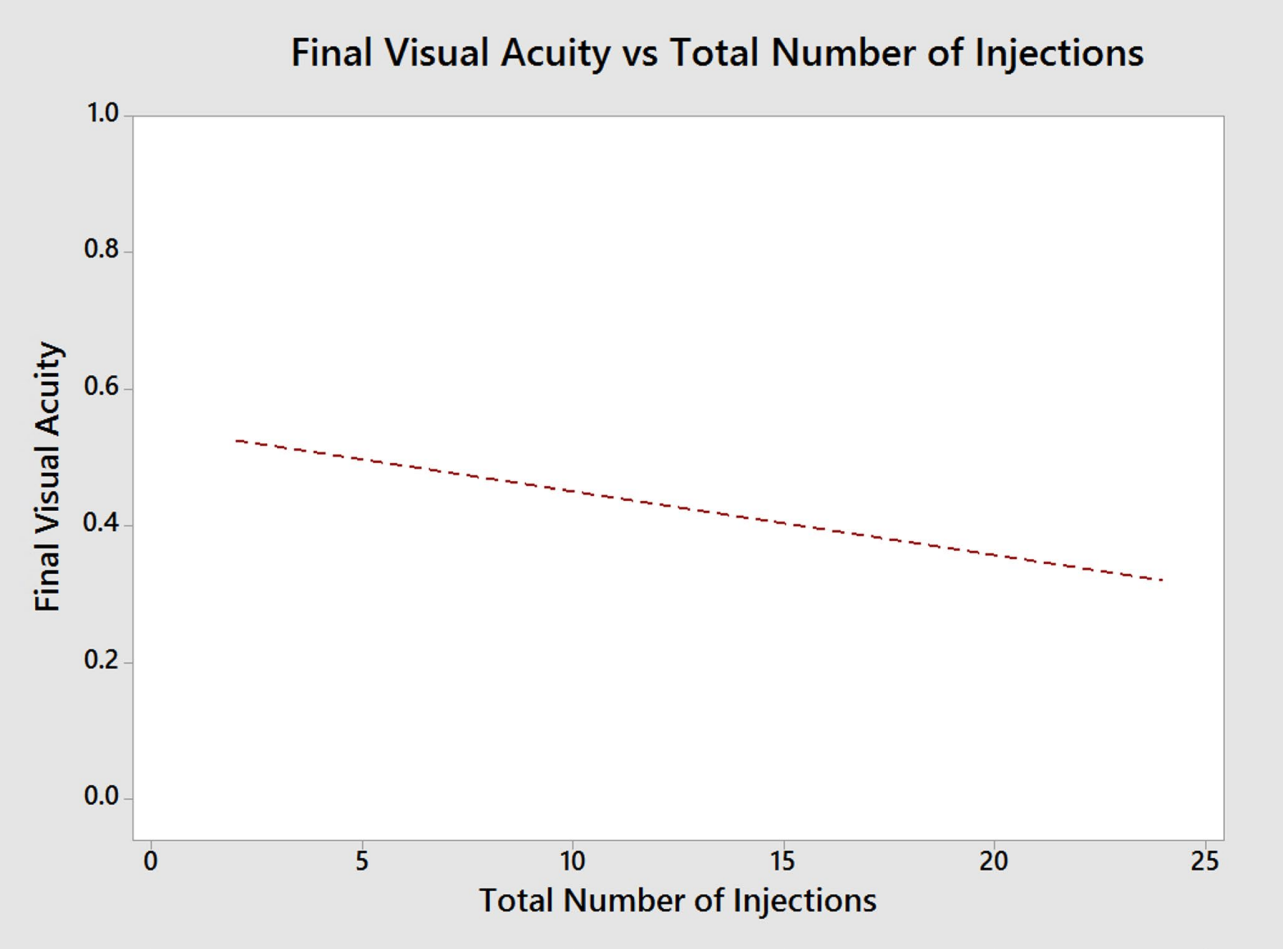
Relationship between final best-corrected visual acuity (BCVA, logMAR) and the total number of intravitreal injections administered during the study period

Figure 3 further visualizes the correlation between BCVA throughout the follow-up and the number of anti-VEGF injections received, demonstrating a trend for BCVA improvement in relation to an increased number of received anti-VEGF injections.

## Discussion

This study evaluated the need for anti-VEGF treatment in treatment-naïve mMNV patients by analyzing 55 eyes that received an initial three monthly injections of bevacizumab followed by a PRN regimen, and assessed their BCVA outcomes over a 24-month period. A significantly higher number of injections were administered during the first year compared to the second year of treatment. The mean baseline BCVA was 20/50 (Snellen equivalent) and remained stable at 20/50 throughout the two-year follow-up. The mean age seemed to be in accordance with the previously reported mMNV age groups 49 [5], 44–50 [6], 55 years old [7], 51–58 [8–11]. While its BCVA seems to be within the upper range of the previously reported baseline VA 20/50 − 20/80 [6], 20/100 [5], 20/160 [7], 20/235. [12] During the follow-up till the end of the study the stability in VA was achieved with a mean injection of 8.78 within the first 12 months and 3.45 within the end of 24 months, this was in contrast to previous reports which demonstrated a relative improvement from baseline VA with fewer amount of injections; Wu et al. [5]. reported VA improvement from 20/100 to 20/50 with mean 3.44 ranibizumab injections at 12 months, Mandal et al. [12] reported improvement in VA from 20/235 to 20/71 with mean 1.6 injections at 6 months, Arias et al. [7]. 20/160 to 20/100 with a mean of 1 injection at 6 months, Parodi et al. [6]. 20/80 to 20/50 with mean 3.8 injections at 24 months, Ruiz-Moreno et al. [13] 20/62 to 20/38, Chan et el [14]. improvement in VA from 20/80 to 20/53 in 6 months, Ladaique at et [15]. 20/63 to 20/40 with 2.2 injections at 24 months.

In clinical practice, the nature of mMNV is considered to be less aggressive than other neovascular diseases (e.g. AMD) and often treated with less aggressive treatment regimes, as PRN [3]. Due to the potential risk of retinal break, retinal detachment, and other complications in these highly myopic eyes and the common clinical practice that most eyes tend to improve after a single injection, many studies adopt the regimen without a loading dose of three consecutive monthly injections as in nAMD [5, 16]. In a report by Ravenstijn et al. [17] of 98 eyes with up to 12 years follow up, Visual acuity initially improved after a median of 2 injections, but was followed by gradual vision loss by the 4-year mark, which seems to align with our results which also emphasize a trend for BCVA improvement following every Anti-VEGF injection, with an eventual decline in BCVA without maintaining this improvement at the end of the follow-up. The suggested superiority of T&E and fixed monthly regimens over PRN may be possibly explained by the rationale that PRN regimens only involve retreatment upon the occurrence of disease activity. This reactive approach may predispose patients to greater fluctuations in retinal thickness in contrast to proactive approaches such as T&E.

The low number of injections given to mMNV patients with the lack of difference in BCVA from baseline throughout follow-up may lead us to the assumption that mMNV patients may be undertreated by having a relatively “loose” PRN injection regimen in real life. Of note, the long-term prognosis of mMNV seems to be very poor without treatment, this was demonstrated in a 10-year follow-up study of mMNV by Yoshida et al. [18] who reviewed 25 cases of mMNV left untreated with VA deterioration to 20/200 in 89% and 96% of eyes in 5 years and 10 years, respectively [19].

A PRN regimen using other agents (ranibizumab, aflibercept) was retrospectively analyzed over a 4-year period, with stratification by pathologic versus non-pathologic myopia [20]. Best-corrected visual acuity (BCVA) outcomes were comparable between groups; however, the non-pathologic myopia group required a higher number of injections and clinical visits. Although our study did not stratify patients based on the degree of pathologic myopia, our findings are consistent with this study, indicating that more frequent injections and visits are necessary to maintain BCVA.

Another study monitored 26 eyes with myopic neovascularization (MNV) secondary to pathologic myopia, treated with either ranibizumab or aflibercept using a pro re nata (PRN) regimen over a 10-year period. The final best-corrected visual acuity (BCVA) remained comparable to baseline [21]. In our cohort, 76% of eyes demonstrated stable BCVA; however, no eyes exhibited visual improvement. These findings may indicate that a higher injection frequency is required to achieve functional gains.

In contrast, recent studies have compared the regimens of PRN and T&E with the opposite conclusion. A retrospective study by Swaminathan et al. comparing the PRN and T&E regimens found that both achieved significant BCVA improvement at 12 months and at the last follow-up, but PRN had more recurrences and required fewer injections. Furthermore, a meta-analysis of randomized controlled trials evaluated two PRN injection regimens differentiated by their retreatment criteria. One regimen employed BCVA stability as the basis for retreatment, whereas the other relied on indicators of disease activity—defined as vision impairment attributable to intraretinal fluid, SRF, or active leakage secondary to pathologic myopia, as determined by optical coherence tomography and/or fluorescein angiography. The analysis demonstrated that disease activity–guided retreatment resulted in a reduced number of injections while achieving comparable visual outcomes to the BCVA stability–guided approach [22]. 

This study offers insight into the real-world application of a PRN protocol for the treatment of mMNV. It highlights the absence of significant BCVA improvement despite a relatively low number of injections. This outcome may be attributed to suboptimal retreatment strategies. Contributing factors may include limited access to medical care at symptom onset, patients’ difficulty in detecting subtle Changes in VA, and potentially prolonged intervals between clinical visits. Limitations of this study involve its retrospective nature, the lack of OCT image analysis or the type of intravitreal injections received. The MD-Clone software enables extraction of data from medical records based on predefined queries, and this approach was used in the present study. However, a limitation of the software is that it cannot extract free-text information but only structured data from predefined fields. Consequently, the analyses were restricted, as information regarding OCT imaging results could not be retrieved for this study. In addition, we did not have data regarding the refraction or axial length. Since some patients were treated using a PRN regimen and others using a TAE regimen, quarterly analysis allowed for a better evaluation of the mean number of injections compared to annual or monthly analyses. The strength of this study involves the relatively long-term follow-up of 2 years based on a quarterly number of injections.

Our findings underscore the lack of significant improvement in best-corrected visual acuity (BCVA) despite a relatively low number of administered injections. This may reflect a common misconception that myopic macular neovascularization (mMNV) is a less aggressive pathology, potentially leading to undertreatment. These results suggest that a more intensive treatment approach—such as a treat-and-extend (T&E) regimen—may be necessary to achieve better visual outcomes compared to a pro re nata (PRN) protocol. Further large-scale randomized controlled trials are warranted to evaluate the efficacy of T&E in this context and to determine whether a shift in the current treatment paradigm is justified.

## Data Availability

The data that support the findings of this study are available from the corresponding author, [RA] upon reasonable request.
